# Intra-observer and inter-observer reliability of leg circumference measurement among six observers: a single blinded randomized trial

**Published:** 2017

**Authors:** Y Bakar, ÖC Özdemir, S Sevim, E Duygu, A Tuğral, M Sürmeli

**Affiliations:** * School of Physical Therapy and Rehabilitation, Abant Izzet Baysal University, Bolu, Turkey

**Keywords:** lower extremity, circumference measurement, inter-observer reliability, intra-observer reliability, reproducibility of results

## Abstract

**Hypothesis:** Circumference measurement of extremities that was reported to be a reliable method as long as being standardized is commonly used both in clinical and home settings by clinicians or caregivers due to its cheapness and easy use.

**Objectives:** The aims of this study were to determine the inter-observer and intra-observer reliability of manual circumference measurement among different observers and various measurement points.

**Methods and Results:** A total of 58 lower limbs were included in the study. Both lower limbs of each subject were assessed by 6 observers randomly using circumference measurement method from 9 reference points specified with a Leg-O-Meter. All observers performed the measurements and they were blind to each other’s measurements.

**Results:** Measurement results from reference points were statistically significant between good to perfect (ICC 0.65-0.99, p<0.001). Interrater reliability of all observers’ first and second measurements showed perfect reliability for both measurements (ICC: 0.92-0.99, p<0.001).

**Conclusions:** This study demonstrated that the lower extremity manual circumference measurement is a reliable method for clinical practice.

**Abbreviations:** BMI = Body Mass Index, ICC = Intraclass Correlation Coefficient, Metatarsal heads (cA), Ankle-heel (cY), Ankle (cB), Distal beginning point of gastrocnemius (cB1), The widest point for gastrocnemius (cC), Head of fibula (cD), Midline of knee (cE), Midline of thigh (cF), Groin level (cG)

## Introduction

Some clinical tools are needed to measure different variables of validating a treatment, performing an adequate follow-up of a disease, or studying an athlete’s performance. This measurement should allow the reproduction of these measurements with an acceptable degree of accuracy and with knowledge of the measurement error [**1**]. It is essential for today’s health care providers to evaluate the patient reliably and monitor the treatment outcomes accurately [**2**]. 

Edema, hypertrophy, and atrophy are commonly encountered clinical manifestations. It is necessary to accurately measure the changes in limb volume to monitor the course of an underlying disease or the effect of treatment [**3**]. Several methods for limb volume assessment are available and each has its own advantages and disadvantages [**2**,**4**,**5**]. The “gold standard” is defined as the volume determined by water displacement [**6**]. However, this method is cumbersome and difficult to use in a clinical setting because of the difficulties in filling and refilling the volumeter tank, the risk of infection and spilling water [**7**]. On the other hand, manual circumference measurement is a reasonably priced, portable, easy to use and clinically practical method, used to estimate limb volume changes from suitable geometric models and mathematical formulas or algorithms [**5**,**8**]. Circumferential measurements are usually made and compared with those for the other limb as a sum or average or as a computed volume of a limb segment [**9**]. Those are used not only to obtain volume calculating from these measurements but they also offer information about the localization of edema and atrophy. In addition, it allows the evaluation of the volume of a specific part of a limb. Determination of edematous limb volumes based on circumferential methods has been reported to be highly correlated with volumes determined by water displacement [**10**]. 

Evidences indicated that circumferential measurement methods are valid and reliable if the method is standardized [**6**,**9**]. At this point, intra-observer and inter-observer reliability play an important role for standardization. Reports showed that measurements at specific intervals between two observers indicated an excellent intraclass correlation coefficient [**11**,**12**].

Physical evaluation routines are usually performed more than once by a single physical therapist or by more than one physical therapist [**13**]. Therefore, it is important for the physical therapists that the measures are reliable within and between therapists [**14**]. However, intra-observer and inter-observer agreements of leg circumference assessment by tape measure have been studied, yet those did not contain more than 3 observers and various circumference points had not been measured [**8**]. Thus, the aims of this study were to determine the inter-observer and intra-observer reliability of manual circumference measurement among different physical therapists and varied measurement points.

## Material and Methods

This study was carried out between April 2016 and May 2016 at Abant Izzet Baysal University School of Physical Therapy and Rehabilitation for evaluating the intra-observer and inter-observer reliability of manual circumference measurement of the lower limbs. The inclusion criteria were determined as being volunteer and older than 18 years. The exclusion criteria were determined as having deformity, contracture, active ulcer, infectious skin disease, surgery, and acute trauma history on the lower limbs, cardiac illness, using corticosteroid and diuretic medications. A total of 90 subjects were informed about the study design and content before the study was started. Out of those, 30 for personal reasons, 3 for health problems, 5 for their work-related reasons and 23 subjects, who refused to take part in the study, were excluded. The study was approved by the Clinical Research Ethics Committee of Abant Izzet Baysal University Faculty of Medicine (IB.30.2.ABÜ.0.20.05.04–050.01.04–60). A verbal and informed consent was obtained from all the subjects included in the study. 

The duration of study, contents of the application and measurement methods were explained to the subjects. Demographic data including age, height, weight, BMI, medical history and family history of the subjects were recorded. The study started with 29 male subjects with a mean age of 23.22 ± 1.38 years. Both lower limbs of each subject were assessed by using circumference measurement, separately.

Before starting the study, all the physical therapists were informed about the details of the measurement protocol. All the observers who were measuring were right-handed. Prior to the circumferential measurements, reference points were specified with a Leg-O-Meter by the senior physical therapist. Leg-O-Meter is a simple and non-invasive tool that objectively helps determining the circumferential measurement points of the lower limb and provides an opportunity to make a comparison between limbs [**11**]. The nine reference points specified for the circumferential measurements of the lower limb were as follows: metatarsal heads (cA), ankle-heel (cY), ankle (cB), distal beginning point of gastrocnemius (cB1), the widest point for gastrocnemius (cC), head of fibula (cD), midline of knee (cE), midline of thigh (cF) and groin level (cG). Subjects were advised to sit on an examination bed while knees were extended. The Leg-O-Meter was placed under the limb whilst ankles were in neutral position and the subjects were asked to maintain the position in which they were placed. A surgical pen was used to mark the reference points. 

**Fig. 1:**
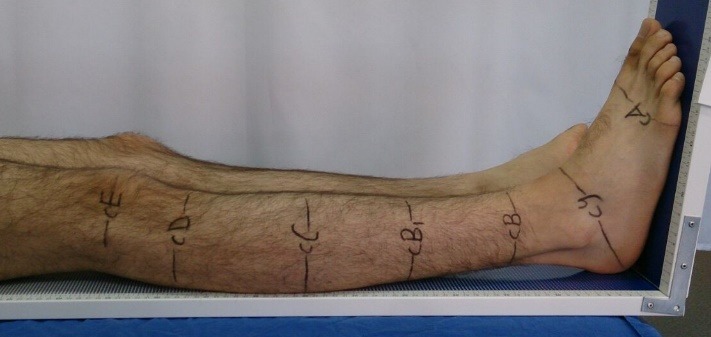
Marked measurement points

**Fig. 2:**
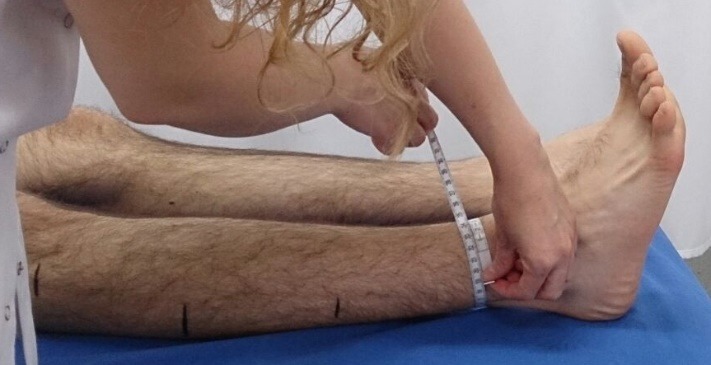
One of the authors measuring the ankle

A standard, non–elastic, bendable tape with a sensitivity level of 0.1 cm, and one-centimeter width was used for the measurements. The tape was enclosured around the limb while the observer was holding the zero end of the tape with the left hand and the other end of the tape with the right hand. Measurement results were observed from the point where the tape intersected with number zero. Particular attention was paid to observe the measurement beside the reference point instead of observing over it. Measurements were recorded in centimeters to achieve the standardization. All observers performed the measurements from the reference points defined previously and they were blind to each other’s measurements. The measurements were repeated one week later at the same time of the day and in the same conditions (positioning, reference points, etc.). Before the second observation, each subject was questioned again, whether having any trauma history or a change in the nutritional habits that could affect the results. In case conditions such as the ones mentioned above occurred, they were excluded. A computerized randomization system was used to queue the observers to avoid any systemic differences between them. An individual who was blinded to the study recorded measurement scores and times of each observer.

## Statistical Analyses

Descriptive analyses were used to calculate the means and standard deviations of the demographic variables and each lower limb was considered individually. The intraclass correlation coefficient (ICC) was used to calculate the intra-observer and inter-observer reliability. The values of the ICC range from 0 to 1, with a higher value indicating better reliability. Intraclass correlation coefficient (ICC) was categorized as poor (<0.40), fair to good (0.40–0.75), and excellent (>0.75). One way Anova was used for the comparison of the measurement times between the observers and the Paired t test was used for the comparison of first and second measurements of the observers. Statistical significance level was accepted as p<0.05. All statistical tests were carried out by using PASW (SPSS Institute, Chicago, IL, USA) (ver. 18).

## Results

A total of 58 lower limbs were included in the study. Mean age and BMI of participants were 23.22 ± 1.39 years and 23.35 ± 3.91 kg/ m2, respectively. Mean and standard deviation values of six observers from the reference points at first and second measurement are shown at **Table 1**. 

**Table 1:** The mean values of the first and second measurements applied at the reference points, made by the six observers

Observer		cA	cY	cB	cB1	cC	cD	cE	cF	cG
		X ± SD	X ± SD	X ± SD	X ± SD	X ± SD	X ± SD	X ± SD	X ± SD	X ± SD
1	First (cm)	24.21 ± 1.03	33.53 ± 1.53	22.14 ± 1.82	24.55 ± 2.08	35.79 ± 3.08	33.63 ± 2.68	37.05 ± 2.79	51.18 ± 5.00	56.98 ± 5.66
	Second (cm)	24.16 ± 1.11	33.54 ± 1.47	22.08 ± 1.76	24.53 ± 2.10	35.65 ± 3.03	33.28 ± 3.08	37.00 ± 2.74	50.74 ± 5.61	56.89 ± 5.56
2	First (cm)	23.94 ± 1.02	33.56 ± 1.56	22.22 ± 2.35	24.75 ± 2.57	35.84 ± 3.09	33.79 ± 2.78	37.06 ± 2.78	50.93 ± 4.75	56.87 ± 5.90
	Second (cm)	24.00 ± 1.18	33.38 ± 2.02	22.00 ± 1.41	24.51 ± 2.11	35.64 ± 3.24	33.67 ± 2.69	37.09 ± 2.70	50.51 ± 5.27	56.69 ± 5.84
3	First (cm)	24.11 ± 1.16	32.98 ± 1.90	22.03 ± 1.43	24.52 ± 2.11	35.72 ± 3.35	33.86 ± 3.69	37.04 ± 2.68	50.46 ± 4.96	57.07 ± 5.66
	Second (cm)	24.19 ± 1.11	33.19 ± 1.49	21.95 ± 1.41	24.35 ± 1.99	35.64 ± 3.19	33.17 ± 2.82	36.70 ± 2.55	50.29 ± 4.93	56.89 ± 5.74
4	First (cm)	24.08 ± 1.17	33.25 ± 1.58	22.14 ± 1.44	24.76 ± 2.59	35.84 ± 3.03	33.47 ± 2.83	37.06 ± 2.82	51.40 ± 4.75	56.36 ± 7.06
	Second (cm)	24.09 ± 1.10	33.33 ± 1.47	22.10 ± 1.45	24.51 ± 2.11	35.71 ± 3.02	33.74 ± 3.81	36.90 ± 2.77	51.31 ± 5.00	56.82 ± 5.56
5	First (cm)	23.61 ± 1.34	32.80 ± 1.46	22.01 ± 1.93	24.08 ± 2.01	35.37 ± 3.11	32.96 ± 2.68	36.31 ± 2.59	50.09 ± 4.86	56.50 ± 5.66
	Second (cm)	23.52 ± 1.30	32.94 ± 1.63	21.77 ± 1.44	24.09 ± 2.58	35.18 ± 3.10	32.78 ± 2.59	36.04 ± 2.57	49.77 ± 4.72	56.29 ± 5.63
6	First (cm)	24.24 ± 1.28	33.46 ± 1.45	22.28 ± 1.43	24.68 ± 2.19	35.81 ± 3.60	33.70 ± 2.77	37.15 ± 2.78	51.32 ± 4.65	57.59 ± 5.47
	Second (cm)	24.19 ± 1.12	33.36 ± 1.61	22.16 ± 1.41	24.67 ± 2.24	35.70 ± 3.45	33.63 ± 2.76	37.51 ± 3.45	51.00 ± 5.02	57.55 ± 5.44
**X ± SD** = Mean ± Standard deviation, **cm** = centimeter										

Intra-observer agreements of leg circumference measurement at the metatarsal heads (CA), ankle-heel (CY), ankle (CB), distal starting point of gastrocnemius (CB1), widest gross point for gastrocnemius (CC), head of fibula (CD), midline of the knee (CE), midline of thigh (CF) and groin level (CG) based on the 9 main reference points are shown in **Table 2**. 

**Table 2:** Intra-observer reliability results of lower limb circumference measurements

	Observer 1		Observer 2		Observer 3		Observer 4		Observer 5		Observer 6	
Experience (year)	19		14		16		5		3		3	
Reference point	ICC	p	ICC	p	ICC	p	ICC	p	ICC	p	ICC	p
cA	0.88	<0.001	0.83	<0.001	0.91	<0.001	0.95	<0.001	0.92	<0.001	0.93	<0.001
cY	0.96	<0.001	0.82	<0.001	0.79	<0.001	0.97	<0.001	0.94	<0.001	0.96	<0.001
cB	0.65	<0.001	0.78	<0.001	0.98	<0.001	0.98	<0.001	0.81	<0.001	0.98	<0.001
cB1	0.99	<0.001	0.89	<0.001	0.98	<0.001	0.90	<0.001	0.89	<0.001	0.98	<0.001
cC	0.99	<0.001	0.94	<0.001	0.99	<0.001	0.99	<0.001	0.99	<0.001	0.99	<0.001
cD	0.94	<0.001	0.98	<0.001	0.75	<0.001	0.82	<0.001	0.99	<0.001	0.99	<0.001
cE	0.99	<0.001	0.98	<0.001	0.99	<0.001	0.99	<0.001	0.98	<0.001	0.77	<0.001
cF	0.92	<0.001	0.95	<0.001	0.98	<0.001	0.99	<0.001	0.99	<0.001	0.92	<0.001
cG	0.99	<0.001	0.98	<0.001	0.99	<0.001	0.89	<0.001	0.99	<0.001	0.99	<0.001
**ICC** = Intraclass Correlation Coefficient, **p**<0.05												

The following results were achieved, after the first and second week measurements of the six observers’ intra-observer reliability was analyzed. Each observer’s first and second measurement results from the reference points were statistically significant between good to perfect (ICC 0.65-0.99, p<0.001). 

Interrater reliability of all observers’ first and second measurements was compared separately and results showed a perfect reliability for both measurements (ICC: 0.92-0.99, p<0.001). Results are shown in **Table 3**.

**Table 3:** Inter-observer reliability results of lower limb circumference measurements

Measurement Points	ICC	p
cA	0.95	<0.001
cY	0.92	<0.001
cB	0.92	<0.001
cB1	0.92	<0.001
cC	0.98	<0.001
cD	0.97	<0.001
cE	0.99	<0.001
cF	0.99	<0.001
cG	0.98	<0.001
**ICC** = Intraclass Correlation Coefficient, **p**<0.05		

First and second measurement times of all observers were compared and second measurement time of every observer was statistically significantly shorter (p<0.05) (**Table 4**). 

**Table 4:** Comparison of first and second measurement times of the intra-observers

	Experience (years)	Time of measurement	X ± SD	p
Observer 1	19	First	84.03 ± 15.99	0.002
		Second	79.90 ± 10.32	
Observer 2	14	First	110.20 ± 50.71	<0.0001
		Second	94.79 ± 18.72	
Observer 3	16	First	121.93 ± 27.35	<0.0001
		Second	102.07 ± 18.59	
Observer 4	5	First	142.07 ± 31.13	<0.0001
		Second	124.96 ± 18.76	
Observer 5	3	First	126.72 ± 30.03	<0.0001
		Second	105.48 ± 17.56	
Observer 6	3	First	120.52 ± 28.09	<0.0001
		Second	107.17 ± 24.24	
Paired t test, **X ± SD** = Mean ± Standard deviation, **p**<0.05				

The clinical experience of physical therapists ranged from 3 to 19 years (mean 9.5 years). Clinical experiences of physical therapists were 19, 14, 16, 5, 3, and 3 years, respectively. First and second measurement times of six observers were compared between observers and the differences were not statistically significant (p>0.05) (**Table 5**). 

**Table 5:** Comparison of differences of measurement times between observers

Time	Experience (year)	X ± SD	P	F
Observer 1	19	4.13 ± 13.24	0.158	1.616
Observer 2	14	15.41 ± 47.17		
Observer 3	16	19.86 ± 18.66		
Observer 4	5	18.57 ± 24.83		
Observer 5	3	21.24 ± 22.48		
Observer 6	3	13.48 ± 17.56		
**X ± SD** = Mean ± Standard deviation, **p**<0.05, **F**: One Way Anova				

## Discussion

This study demonstrated that the lower extremity manual circumference measurements applied at different reference points were highly consistent among all the observers in their repetitive measurements and between the observers. The high ICC values found in our study indicated that the deviation was low between the inter-observer and intra-observer measurements. In addition, it was suggested that the reproducibility of the manual circumference measurement method was quite good for the measurement of the lower extremity circumference.

The evaluation of the clinical cases and the examination of the changes in the course of the disease were necessary to determine the severity of the illness or the symptoms and the effectiveness of the treatment. Ensuring a minimal error rate in the measurements is important in terms of obtaining consistency of evaluations and interpretation of the results [**15**]. The best way to ensure consistency between the assessments is that the same person makes the measurements. However, this is not always possible. In such situations, it is important to be consistent with other measures that are conducted by different observers.

Manual circumference measurements are widely used to evaluate symptoms and parameters such as edema, muscle atrophy, obesity, breathing depth. Their advantages are being simple to implement and feasible in residential areas (residential areas, outpatient conditions), easily repeatable, noninvasive and applicable by the patient himself [**16**]. Leg circumference measurement is convenient for routine use in terms of being easy to use and cheaper in the clinic and hospital. Although there is some evidence that stated that optoelectronic techniques are superior to manual tape measurements, it was reported that leg circumference measurements, which are done by spring tape, demonstrated a good correlation with the water displacement method (r = 0.91) and optoelectronic volumeter (r = 0.95). In addition to achieving volume measurements with manual circumference measurements, the comparison of limb measurements with each other provided information on the shape of the limbs and the location of the edema [**17**]. 

Circumferential measurements produce minimal differences between physiotherapists if applied according to proper techniques. It was also reported that compared to water displacement volumeter, the circumferential measurement has better reliability [**18**]. A previous study by Tunç et al. [**8**] revealed that lower limb circumferential measurements from three reference points (medial malleolus, tuberosity of the tibia and patella) showed high-level inter-observer reliability. They also noted that circumferential measurements around tuberosities of the tibia were more reliable than the other reference points. Campagna et al. [**19**] stated that the circumferential measurements from wrist showed high-level reliability between observers (ICC ≥ 0.96).

In this study, the intra-observer reliability values were higher than 0.75 and sufficient, also requisite reliability except for the one of the observer’s measurement from reference point cB. We thought that the reliability of the observers’ measurements from reference point cB were low because, at that point, the limbs shape was more like a cone, the tape having a tendency to slip down and the circumferential measurement score was low which effected the statistical outcomes more than the other reference points. The ICC scores at reference point cA were significant and reliable but the values were lower. The reason for that may be the measurements achieved from different sides of the bone projections. The ICC scores were higher than 0.83 and adequately reliable. Reference point cC was the most coherent point for the observers (ICC=0.94-0.99). This point is the widest point for the gastrocnemius muscle and contains minimum subcutaneous fat, so the observers were unable to squeeze the tape involuntarily. Unlike Tunç et al. [**8**], our ICC scores from the reference point cE, which indicated the knee area between 0.98 and 0.99 except for one observer. We thought that the soft tissue of the knee regions was relatively thin, that being the reason why reliability scores were high.

Wang et al. [**20**] investigated the reliability of circumferential measurement of waist and hip between the observers, stating the reliability at an acceptable level, even the one of the observer who was inexperienced (ICC≥0.95). This result indicated that after required training and standardized procedures, circumferential measurement is a practicable method. We standardized the measurement method by teaching the observers about handling the tape, measuring with tape, and using the Leg-O-Meter before the measurements. In our study, inter-rater reliability scores were higher than 0.90 and reliability between observers was perfect. We thought that the standardization before the measurements inhibited the differences that might originate in the variety of experience and, as a result, reliability was high between all observers. Reliability at the reference points cB and cB1 were at some degree lower than the other points. The reason for that was thought to be the conic shape of the limbs distal reference points, a small shift during the measurement causing a difference. In addition, the gastrocnemius muscle started to widen after reference point cB1, which might have made it harder for the measuring point to be specified. 

At the clinic, it is essential to evaluate the disease for the assessment of treatment achievements and to determine the therapy goals. Using the time effectively is essential especially for the populous clinics [**15**]. It was shown that the second measurement times of the observers were significantly shorter in comparison with the first measurements. We suggested that the reason for this result was the experience of the observers on lymphedema therapy as they used circumferential measurements on their daily routine. The observers’ first and second measurement time differences were minimum for the first two observers. These two observers were the most experienced lymphedema therapists and their working time as physical therapists was longer than the rest of the observers.

## Conclusion

Until present, this has been the first study carried out with six observers, which focused on circumferential measurements intra-observer reliability. One of the strengths of our study was a variety of work experience among observers, the reliability of this study still being high. Another strength of our study was that all the subjects were male, which minimized the effects such as menstrual cycle, hormonal changes or wearing high heels shoes on measurements during the one-week period. We suggested that further studies should be conducted with larger samples and in both genders. Circumferential measurements take place on examining the diseases characterized with edema, especially lymphedema or chronic venous insufficiency [**15**]. We focused on healthy subjects but, for further studies, it is advisable to repeat the study with edematous patients to clarify the clinical usage.

**Source of Funding **

The authors received no financial support for the research, authorship, and/ or publication of this article. 

**Disclosures**

The authors declare that there is no conflict of interest. 

## References

[1] Allington NJ, Leroy  N, Doneux  C (2002). Ankle joint range of motion measurements in spastic cerebral palsy children: intra-observer and inter-observer reliability and reproducibility of goniometry and visual estimation. J Pediatr Orthop B.

[2] Mayrovitz HN, Sims  N, Macdonald J (2000). Assessment of limb volume by manual and automated methods in patients with limb edema or lymphedema. Adv Skin Wound Care.

[3] Roeckaerts F, Vanden Bussche G (1980). Double-blind placebo-controlled studies with flunarizine in venous insufficiency. Angiology.

[4] Czerniec S, Ward L, Refshauge KM, Beith  J, Lee MJ, York S, Kilbreath SL (2010). Assessment of breast cancer-related arm lymphedema—comparison of physical measurement methods and self-report. Cancer Invest.

[5] Ng M, Munnoch A (2010). Clinimetrics of volume measurement in upper limb LE. J Lymphoedema.

[6] Sander AP, Hajer NM, Hemenway K, Miller AC (2002). Upper-extremity volume measurements in women with lymphedema: a comparison of measurements obtained via water displacement with geometrically determined volume. Phys Ther.

[7] Smoot BJ, Wong JF, Dodd MJ (2011). Comparison of diagnostic accuracy of clinical measures of breast cancer–related lymphedema: area under the curve. Arch Phys Med Rehabil.

[8] Tunc R, Caglayan-Tunc  A, Kisakol  G, Unler  GK, Hidayetoglu T, Yazici H (2007). Intra-observer and inter-observer agreements of leg circumference measurements by tape measure based on 3 reference points. Angiology.

[9] Taylor R, Jayasinghe  UW, Koelmeyer  L, Ung  O, Boyages  J (2006). Reliability and validity of arm volume measurements for assessment of lymphedema. Phys Ther.

[10] Meijer R, Rietman  J, Geertzen J, Bosmans  J, Dijkstra P (2004). Validity and intra-and inter-observer reliability of an indirect volume measurements in patients with upper extremity lymphedema. Lymphology.

[11] Bérard A, Kurz  X, Zuccarelli F, Abenhaim  L (2002 ). Validity of the Leg-O-Meter, an instrument to measure leg circumference. Angiology.

[12] Tidhar D, Armer  JM, Deutscher D, Shyu  C-R, Azuri J, Madsen  R (2015 ). Measurement Issues in Anthropometric Measures of Limb Volume Change in Persons at Risk for and Living with Lymphedema: A Reliability Study. J Pers.

[13] Elveru RA, Rothstein JM, Lamb RL (1988 ). Goniometric reliability in a clinical setting. Phys Ther.

[14] Nunes GS, Bayer GS, da Costa LM, de Noronha M (2012 ). Intra-observer and inter-observer reliability of a method to measure ankle plantar-flexion range of motion in the hook-lying position. J Sport Rehabil.

[15] Guex J, Perrin M (2000 ). Edema and leg volume: methods of assessment. Angiology.

[16] Pani S, Vanamail P, Yuvaraj J (1995 ). Limb circumference measurement for recording edema volume in patients with filarial lymphedema. Lymphology.

[17] Stanton A, Badger C, Sitzia J (2000 ). Non-invasive assessment of the lymphedematous limb. Lymphology.

[18] Deltombe T, Jamart J, Recloux S, Legrand C, Vandenbroeck N, Theys S, Hanson P (2007 ). Reliability and limits of agreement of circuferential, water displacement, and optoelectronic volumetry in the measurement of upper limb lymphedema. Lymphology.

[19] Campagna G, Zampetti S, Gallozzi A, Giansanti S, Chiesa C, Pacifico L, Buzzetti R (2016 ). Excellent intra and inter-observer reproducibility of wrist circumference measurements in obese children and adolescents. PloS one.

[20] Wang C-Y, Liu M-H, Chen Y-C (2010 ). Intrarater reliability and the value of real change for waist and hip circumference measures by a novice rater. Percept Mot Skills.

